# Comparative evaluation of aqueous *Beta vulgaris* extract as a natural microbial dye relative to standard stains through image processing

**DOI:** 10.1038/s41598-025-23508-8

**Published:** 2025-11-13

**Authors:** Sheetal Gouda, Navya Shetty, H. A. Arundathi, V. N. Venkatesh, Sooraj Mohan, P. Dinesha

**Affiliations:** 1 Department of Microbiology, Karwar Institute of Medical Sciences, Karwar, 581301 India; 2 Department of Mechanical and Industrial Engineering, Manipal Institute of Technology, Manipal Academy of Higher Education, Manipal, 576104 India

**Keywords:** Beta vulgaris, Natural dye, Sustainable, Microbial diagnostics, Microscopic image analysis, Biological techniques, Biotechnology, Microbiology

## Abstract

The growing environmental concerns about synthetic dyes have increased interest in finding natural, non-toxic alternatives for microbiological staining. This study assesses the effectiveness of aqueous *Beta vulgaris* (beetroot) extract as a staining agent for microorganisms, comparing its performance with traditional dyes. Microbial smears containing bacteria and fungi were prepared using both standard dyes and *Beta vulgaris* extract. Microscopic images were taken and analyzed quantitatively, using measures such as sharpness, entropy, contrast, signal-to-noise ratio, and edge intensity. While statistical analysis with the Wilcoxon Signed-Rank test showed no significant differences in most parameters (*p* > 0.05), the *Beta vulgaris* extract showed clear advantages in fungal staining, providing better morphological detail. However, bacterial staining was less sharp visually. These results suggest that aqueous *Beta vulgaris* extract has potential as a sustainable alternative for specific microbial staining, especially for identifying fungi, though further refinement and broader microbial testing are needed.

## Introduction

Staining is one of the ancient techniques and continues to remain a vital part of diagnostic laboratories. The majority of the stains used today are chemically synthesized and non-biodegradable^1^. They pose a hazardous effect on the environment. The mutagenic and carcinogenic potential^2^ of these dyes are raising alarm about their usage in routine purposes. In this regard, interests are shifted in exploring natural dyes, which are non-hazardous, economical, and environmentally friendly, that critically aim to address the United Nations’ sustainable development goals (3, 6, 12, 13, and 14)^3^.

Natural dyes can be extracted from various plants, animals, minerals, fungi, and bacteria. Among them, plants contribute the highest sources for dyes^4^. Many plants, like indigo^5^, onion peels^6^, annatto seeds^7^, hibiscus^8^, henna^9^, including *Beta vulgaris* (beetroot)^10^, have been investigated in the literature for their ability to dye. The beetroots are easily available and widely grown across the globe. The red colour of beetroot extract is due to the pigment betalains, which consist mainly of betacyanins (75–95%) and betaxanthins (5–25%)^11^. The activity of pigment is better at pH ranging from 3 to 7^12^, and temperature below 50 °C. The extract is also stable for a month at 4 °C, and a year when stored at −30 °C^13^. In addition to imparting colour, beetroot is also known to have anti-inflammatory and antibacterial properties^14^, which suggests we explore its potential as a staining agent. Many studies have proved the ability of the ethanolic extract of beetroot for staining Helminthes^15^. The adult worms of Platyhelminthes and Nematodes are stained effectively. The eggs of Nematodes were darkly stained, making them difficult to identify. Very few articles suggesting the staining ability of beetroot extract on bacteria have been reported in the literature. Anita Oktari et al. showed application of ethanolic extract of beetroot at various concentrations of 5%, 7.5%, 10% and 12.5% as counterstain instead of 0.25% safranin in performing the Gram staining procedure^16^. The Mann-Whitney U test compared the staining efficacy of beetroot extract and safranin and suggested that beetroot extract at 10% and 12.5% was as efficient as 0.25% safranin in performing the Gram staining procedure. Another study by Sutradhar and Bhattacharya used citric acid solvent to extract beetroot pigment^17^. This extract was mixed with glycerol and used for wet mount preparation of fungal slides instead of Lacto phenol cotton blue (LPCB). Based on the visual findings, they concluded that the intensity of colour, contrast, and clarity of fungal structures was as good as LPCB. However, no image processing or statistical analysis was applied in their work.

Despite the scarcity of literature on the staining potential of *Beta vulgaris* extract for specific microbial groups, no comprehensive study has evaluated its efficacy across diverse microorganisms, including bacteria and fungi. The existing literature predominantly focuses on individual microbial types, with no unified approach to assess their broad-spectrum applicability. Moreover, prior studies have primarily utilized organic solvents for extraction, leaving the efficacy of simple aqueous extraction underexplored. Critically, to the best of the authors’ knowledge, there are no documented studies employing objective image-based analysis or digital microscopy metrics to assess the staining performance, neither for *Beta vulgaris* nor for other natural dyes. Hence, this work addresses these crucial gaps by employing an environmentally friendly aqueous extraction method and applying quantitative image analysis to evaluate the staining outcomes in comparison to conventional synthetic dyes. The study outcomes are twofold: promotion of sustainable staining alternatives and evaluation of microscopic images through image analytics.

## Methods and methodology

### Study design and data collection

The study type selected was a prospective, paired, observational design conducted over a period of 2 months. The experimental study was conducted at the Department of Microbiology, Karwar Institute of Medical Sciences, Karwar, India, after obtaining permission from the Institutional Ethical Committee.

### Extraction and purification of crude beetroot dye

500 g of fresh beetroot was procured from the local market of Karwar and washed thoroughly with water to remove visible dirt. The skin was peeled, and the pulp was ground with a mortar and pestle. Around 200 g of ground beetroot was mixed with 100 mL of distilled water in a rotary shaker and incubated in a water bath at 40 °C for 1 h. This was further centrifuged at 3000 rpm for 15 min until all sediments settled at the bottom. The red coloured supernatant is collected and filtered through Whatman filter paper Grade 1. The filtrate thus obtained was used as a dye for staining microorganisms.

### Staining methods

#### Simple stain

Pure cultures of *Staphylococcus aureus* (cocci) and *Escherichia coli* (bacilli) are taken from the stock culture of the Bacteriology laboratory, Karwar Institute of Medical Sciences. Simple staining was performed on a mixture of cocci and bacilli with safranin for 1 min, following the standard procedure^18^. This acts as a control. The test samples containing a mixture of cocci and bacilli are stained with beetroot extract for 1, 3, and 5 min.

#### Gram stain

Gram stain was performed using crystal violet, Gram’s iodine, acetone, and safranin following standard protocol^18^. This was labelled as control. The beetroot extract was used as a counterstain instead of safranin for performing the Gram stain procedure. The duration of application of the extract was varied from 1, 3, and 5 min. The efficacy of staining was compared with the control in terms of clarity of morphology, colour intensity, and contrast.

#### Performing fungal stain

The fungal slants of *Candida spp*., *Aspergillus niger*, *Aspergillus fumigatus*, *Rhizopus spp*., *Trichophyton spp.*, are obtained from the stock culture of the Mycology section of the Microbiology department. Simple staining using crystal violet was performed for Candida spp., negative staining using India ink for *Cryptococcus spp*., and LPCB mount for molds, following the standard procedure^18^. The beetroot extract was used instead of crystal violet, India ink, and LPCB for staining fungi.

### Processing the microscopic images

#### Image preparation and preprocessing

Microscopic images of stained microorganisms were captured under standardized illumination and magnification settings. Each microorganism was imaged using two staining protocols, one with a conventional (standard) dye and another with a test dye (*Beta vulgaris*). All images were acquired in high-resolution (at least 300 dpi) camera and exported in lossless grayscale format (8-bit) to ensure consistent intensity distribution and prevent compression artifacts.

Before analysis, images were subjected to preprocessing steps to minimize artifacts introduced by the imaging system. Specifically, wherever present, a microscope-embedded marker was computationally detected using the Hough line transform and suppressed using a binary masking approach. This ensured that metrics derived from the image represented intrinsic structural and textural attributes of the specimen rather than external overlays or borders.

#### Quantitative image quality metrics

To objectively evaluate and compare the clarity and structural representation of the microorganisms, five image-based quantitative parameters were computed using established image processing algorithms implemented in Python (OpenCV, NumPy, and Scikit Image libraries). The code developed was executed using the Google Colab interface.

##### Sharpness

The sharpness of each image was estimated by calculating the variance of the Laplacian operator applied to the grayscale image. This measure quantifies the spatial frequency of edges, whereby higher variance values indicate a greater concentration of fine details and well-defined cellular boundaries. The sharpness is calculated as shown in Eq. (1)

1 $$\:S=var\:\left({\nabla\:}^{2}I\right)$$

Where, $\:{\nabla\:}^{2}I$ is the Laplacian operator applied to the image $\:I$.

##### Entropy

Shannon entropy was computed to evaluate the randomness and complexity of pixel intensity distribution. Entropy reflects the amount of information embedded within the image; higher values imply a broader distribution of intensities and hence greater textural complexity, potentially indicating enhanced staining differentiation and microbial morphology. Derived from information theory that reflects the complexity or randomness of the intensity distribution, a high entropy value indicates a more heterogeneous image with diverse grayscale intensities, which is often desirable in biological images where subtle textural differences are important. Lower entropy suggests uniformity or overexposure, both of which may result in loss of diagnostic information. The Shanon Entropy, $\:H$ is calculated using Eq. (2)

2 $$\:H=-\sum\:_{i=0}^{L-1}{p}_{i}{\text{log}}_{2}\left({p}_{i}\right)$$

Where, ​$\:{p}_{i}$ is the probability of occurrence of the $\:i$^th^ intensity level and $\:L$ is the number of gray levels (256 for 8-bit images).

##### Contrast

Contrast was quantified as the standard deviation of grayscale intensity values across the entire image as calculated using Eq. (3).

3 $$\:C=\sqrt{\frac{1}{N}\sum\:_{j=1}^{N}{({I}_{j}-\mu\:)}^{2}}$$

Where, $\:{I}_{j}$ represents the intensity of $\:j$^th^ pixel, $\:\mu\:$ is the mean intensity and $\:N$ is the total number of pixels.

This measure captures the dynamic range of tonal variations and serves as a global descriptor of how prominently different regions of the microorganism are visually distinguishable from the background. Mathematically, it is computed as the standard deviation of pixel intensities from the mean. A high contrast value implies significant variation between bright and dark regions. Although it depicts improved visual detectability of structural features such as cellular boundaries, it is a global descriptor and does not account for local variations.

##### Signal-to-noise ratio (SNR)

SNR was calculated as the ratio of the mean pixel intensity, $\:\mu\:$ (signal) to the standard deviation, $\:\sigma\:$ (noise) within the image (Eq. (4)). A higher SNR implies that the signal (i.e., microbial features) is more distinctly resolved from the inherent background noise, suggesting better image clarity and less variability due to artifacts.

4 $$\:SNR=\frac{\mu\:}{\sigma\:}$$

##### Edge intensity

Edge intensity quantifies the strength of edges across the image and is particularly useful for assessing structural clarity. The Sobel operator calculates the gradient of the image in two orthogonal directions, highlighting regions with rapid intensity changes. The magnitude of the gradient vector indicates the prominence of each edge. By averaging these magnitudes, we obtain a global descriptor that reflects the overall visibility of structural features in the image. High values suggest sharper, better-defined contours. This metric captures the average strength of edge transitions across the image and reflects the ability of the dye to reveal well-defined cellular boundaries or structural discontinuities. The edge intensity is calculated using Eq. (5).

5 $$\:E=\frac{1}{N}\sum\:_{i=1}^{N}\sqrt{{\left({{\nabla\:}^{i}}_{x}\right)}^{2}+{\left({{\nabla\:}^{i}}_{y}\right)}^{2}}$$

$\:{{\nabla\:}^{i}}_{x}$ and $\:{{\nabla\:}^{i}}_{y}$ are the gradients in the $\:x$ and $\:y$ directions of the pixel $\:i$, and $\:N$ being the total number of pixels.

#### Data processing and output

Each image was independently analyzed, and metric values were computed programmatically. Results were compiled into a structured data table, enabling direct numerical comparison between images stained with the standard and test dyes. This quantitative approach supports the objective assessment of staining efficacy and morphological clarity in microscopic imaging.

To further analyze the output of the data, a hypothesis was formulated using the Wilcoxon Signed-Rank Test to ascertain if there were any significant deviations between the test dye and the beetroot dye concerning the various image quality metrics. The Wilcoxon Signed-Rank Test was used, considering the lower sample size and a non-normal data distribution. At a 95% confidence level, a probability value less than 0.05 was considered statistically significant.

## Results

The beetroot extract obtained was bright red in color, with a pH of 5.5. It was stored at 4 °C in amber coloured bottle till completion of the experiment. The extraction of beetroot pigment can be done by various methods. Though the newer methods like ultrasound-assisted extraction and microwave-assisted extraction processes yield higher pigment, the procedure is often expensive and complicated to be carried out in routine microbiological laboratories. There is not much statistical difference in extraction between newer methods and the solvent extraction method^19^. Since the aqueous extraction is simple, easily carried out in resource-limited settings, this method of extraction was followed. The temperature above 50 °C causes degradation of the pigment; hence, the extraction in this study was carried out at 40 °C. The extract obtained in the study has a pH of 5.5, suggesting a clear compliance, as the biological activity of the pigment is generally demonstrated in the pH range of 3 to 7.

### Simple stain

**Fig. 1:**
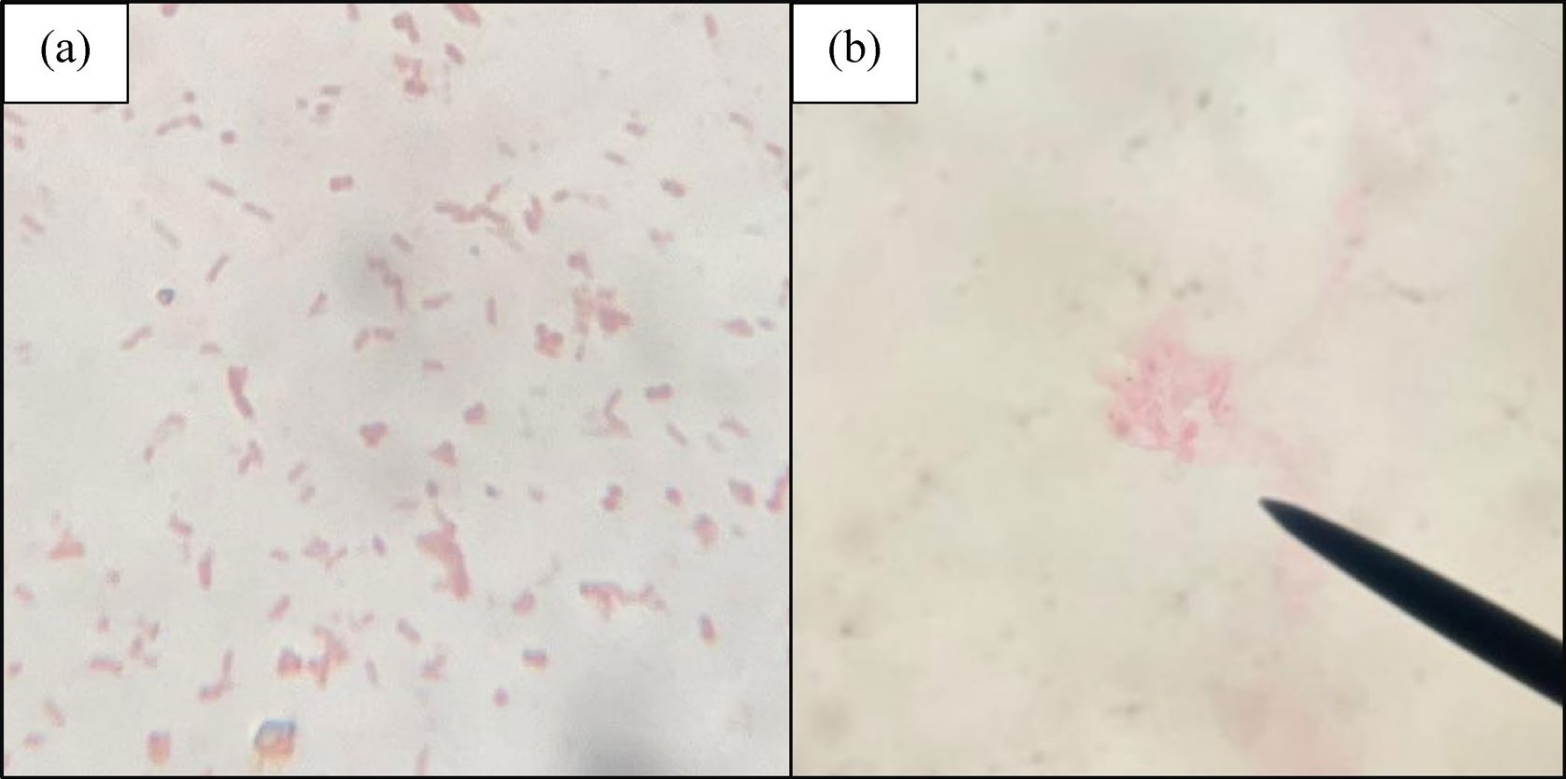
Comparison of simple staining with (**a**) Safranin for 1 min and (**b**) *Beta vulgaris* for 5 min.

A simple stain of a mixture of *Staphylococcus aureus* and *Escherichia coli* was performed using beetroot extract. Bacteria were poorly stained even after the application of the dye for 5 min. The staining of bacteria with 5 min of beetroot extract is compared with 1 min staining of safranin and presented in Fig. 1.

### Gram stain

**Fig. 2:**
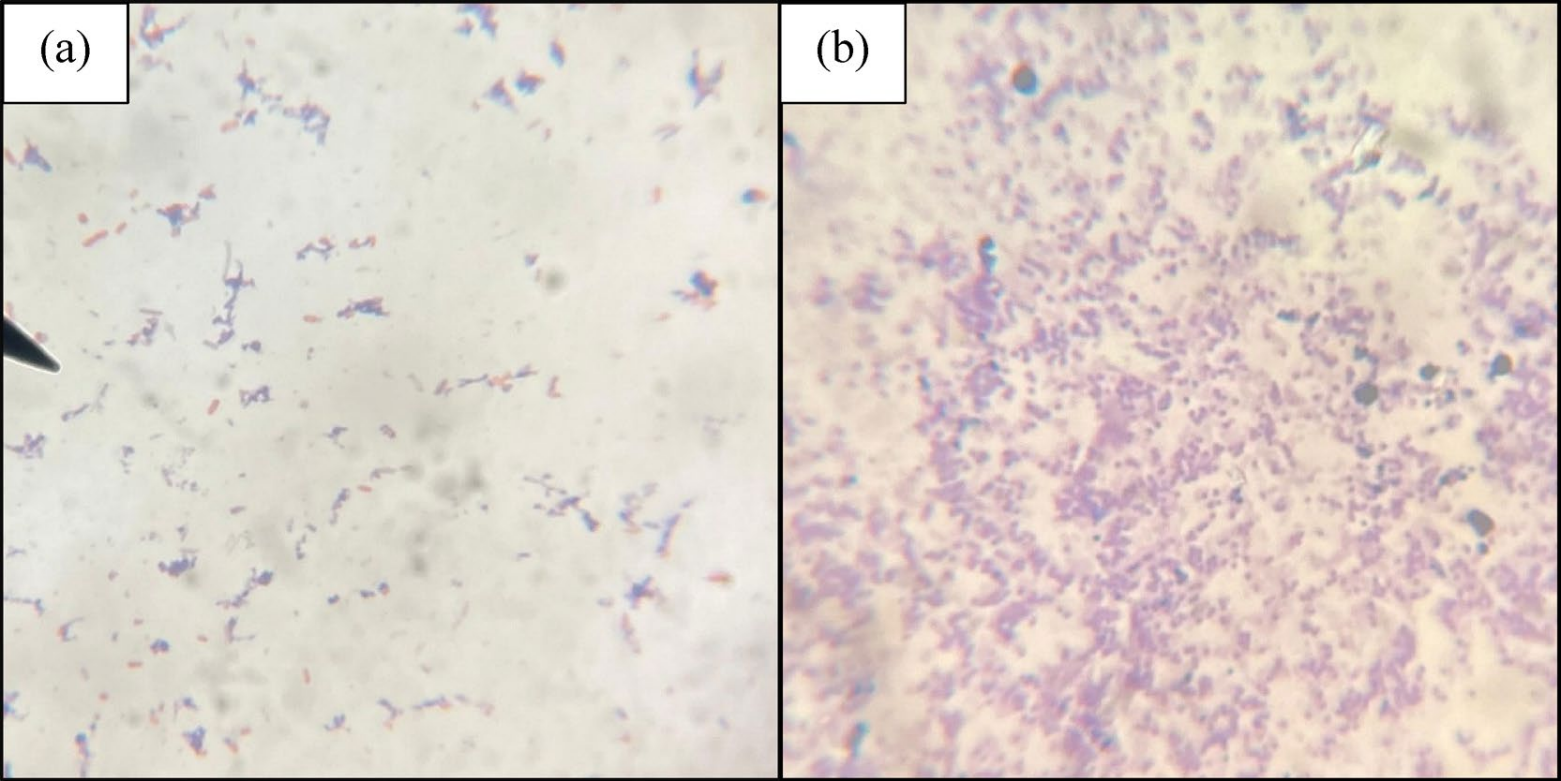
Comparison of gram staining with counterstain of (**a**) Safranin applied for 1 min and (**b**) *Beta vulgaris* applied for 5 min.

Gram-positive cocci appeared purple, and Gram-negative bacteria appeared pink on using beetroot extract as a counterstain in the Gram staining procedure. However, the clarity and contrast of counterstain (beetroot extract) were poor even at the end of 5 min when compared to the 1-minute staining of safranin, as shown in Fig. 2.

### Fungal stain

**Fig. 3:**
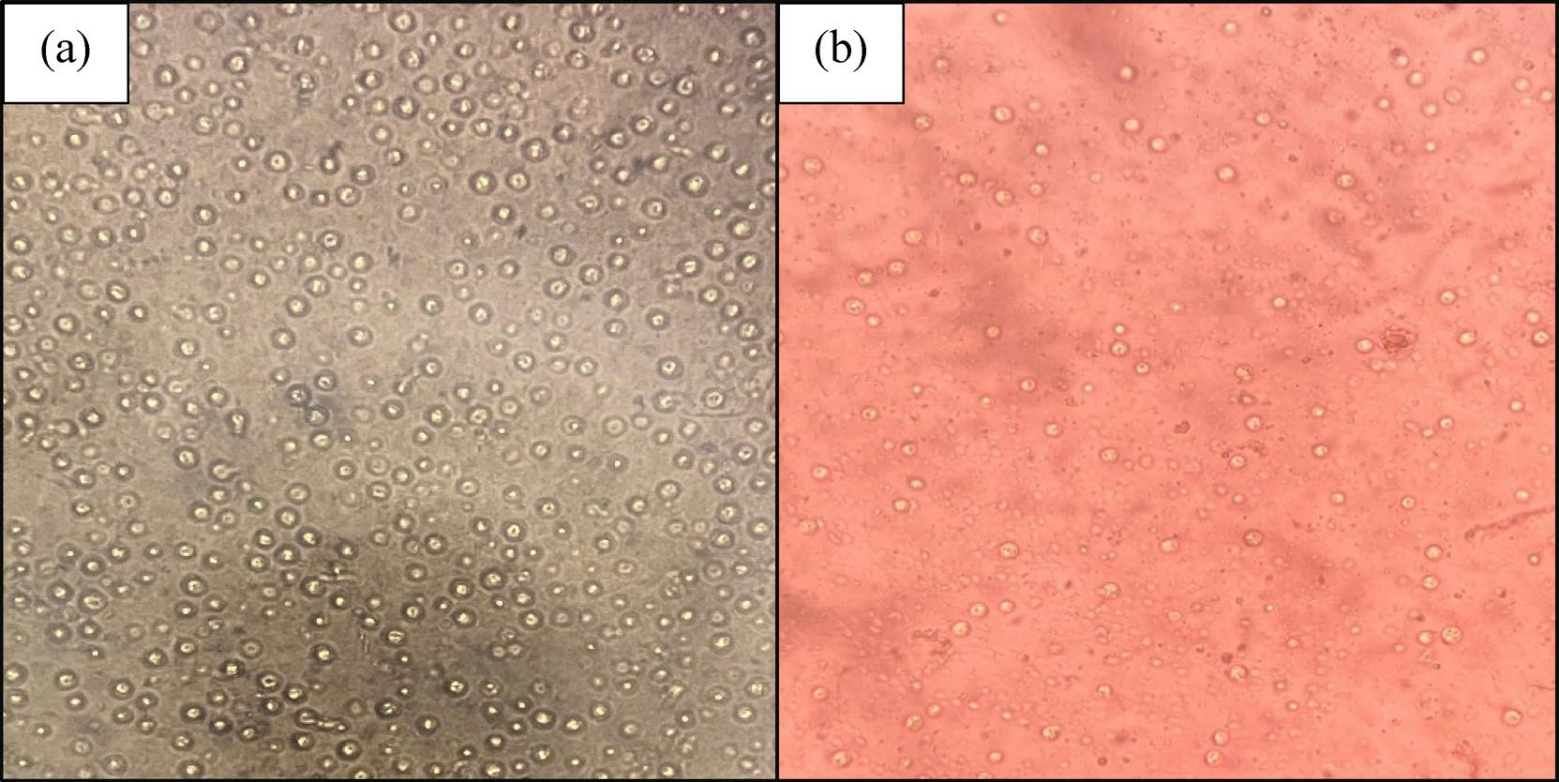
Comparison of capsular staining of *Cryptococcus spp*. with (**a**) India ink and (**b**) *Beta vulgaris*.

The beetroot extract could not stain *Candida spp.*, but the capsule of *Cryptococcus spp.* was demonstrated. The capsular staining of *Cryptococcus spp*. was comparable to India ink preparation, as shown in Fig. 3.

**Fig. 4:**
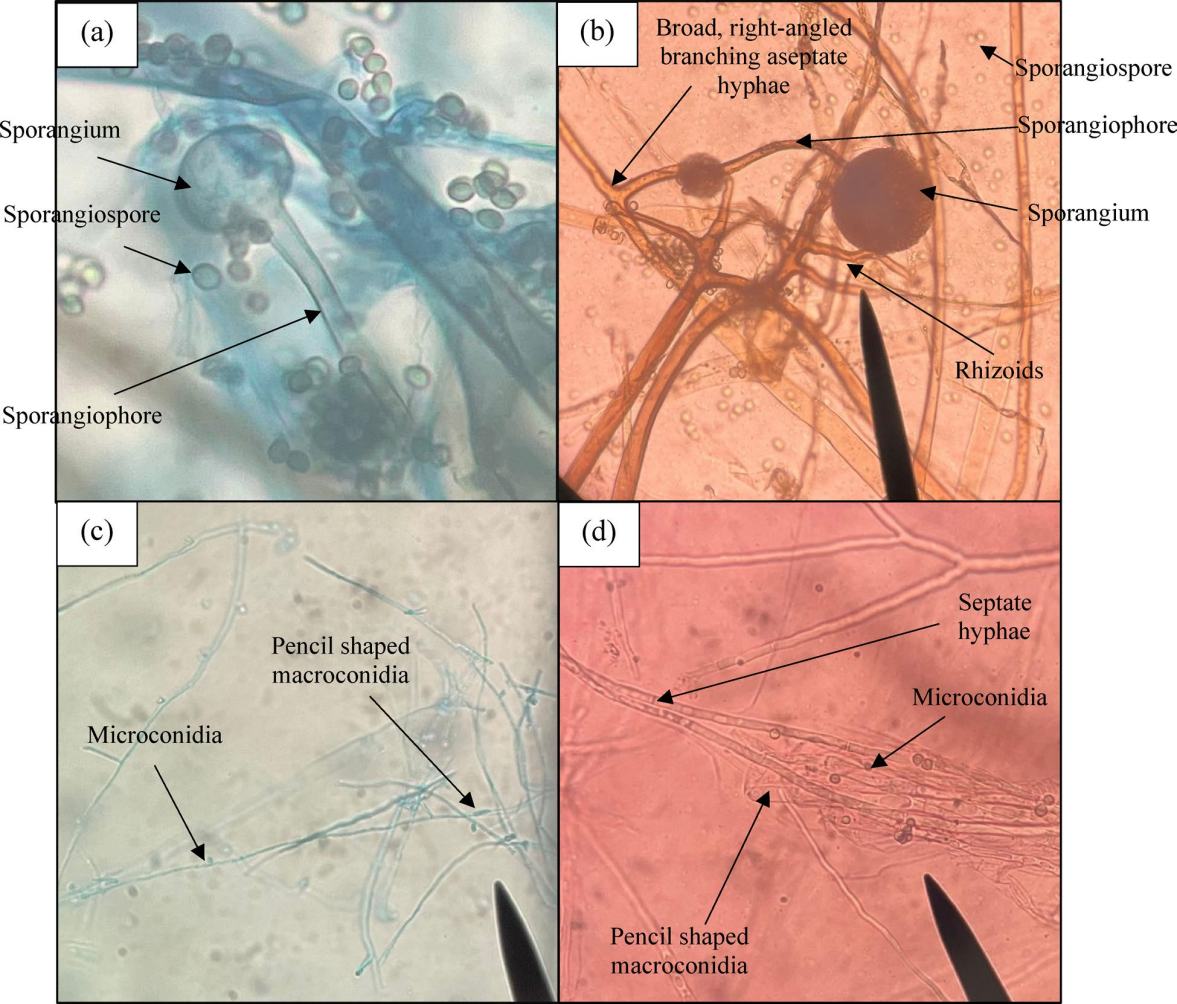
Comparison of (**a**) *Rhizopus spp*. stained with LPCB dye and (**b**) *Rhizopus spp*. stained with *Beta vulgaris* (**c**) *Trichophyton mentagrophytes* stained with LPCB dye and (**d**) *Trichophyton mentagrophytes* stained with *Beta vulgaris*.

**Fig. 5:**
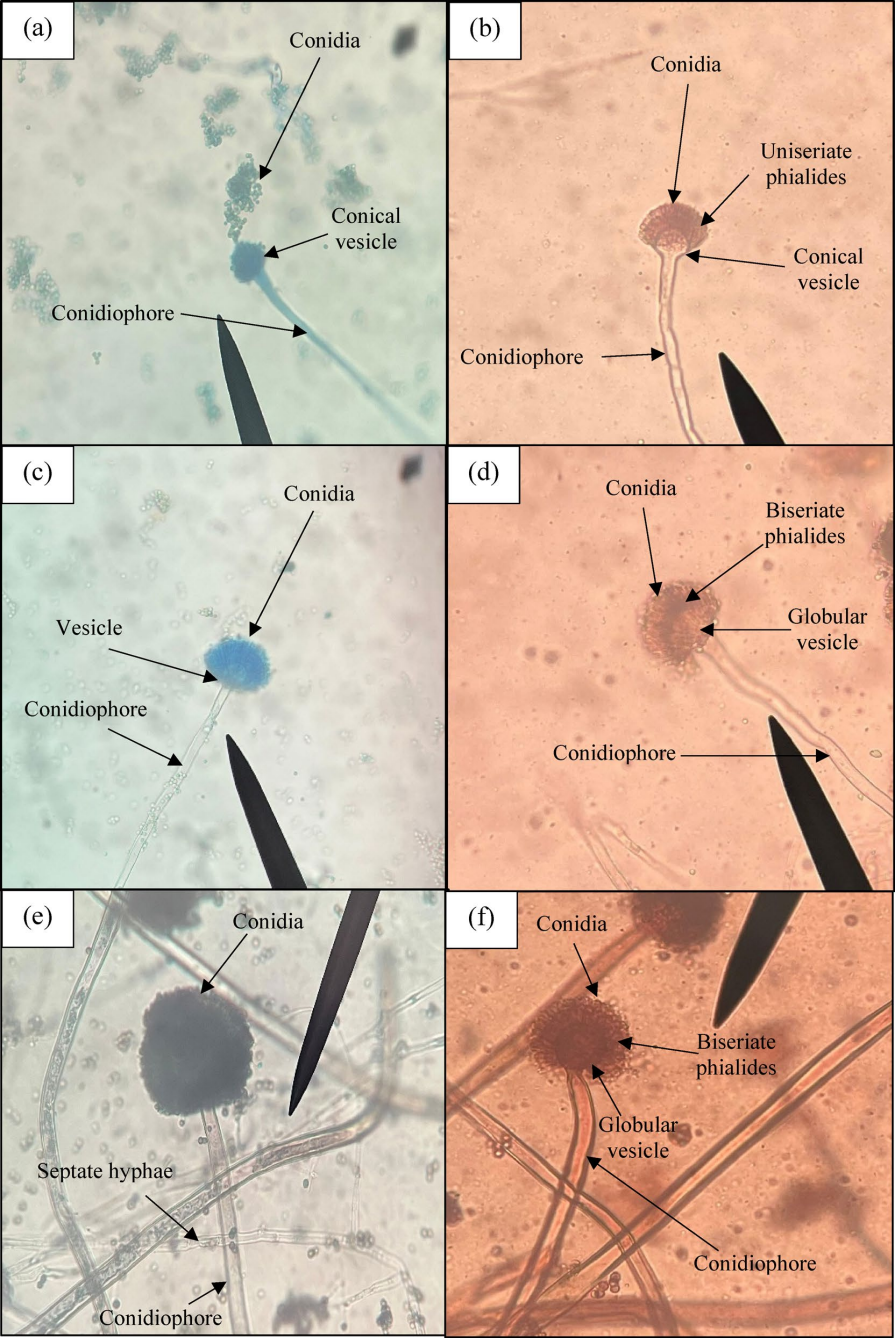
Staining comparison of (**a**) *Aspergilius fumigatus* with LPCB dye and (**b**) *Aspergilius fumigatus* with *Beta vulgaris* (**c**) *Aspergillus flavus* with LPCB dye and (**d**) *Aspergillus flavus* with *Beta vulgaris* (**e**) *Aspergillus niger* with LPCB dye and (**f**) *Aspergillus niger* with *Beta vulgaris*.

The staining ability of beetroot extract for staining *Rhizopus spp*. is presented in Fig. 4 (b) in comparison with that of the LPCB dye, as shown in Fig. 4 (a). The ribbon-like hyphae, rhizoids, sporangium, and sporangiospore are clearly seen. For *Trychophyton spp.*, septate hyphae, microconidia, and macroconidia can be appreciated. The comparative image of staining Dermatophytes by LPCB and beetroot extract is shown in Fig. 4 (c) and (d). For staining *Aspergillus spp*. the septa, conidia, arrangement of metullae, sterigmata, and conidiophores are clearly demonstrated. The clarity and contrast were much better compared to routine LPCB staining, as shown in Fig. 5 (a)-(f). The morphological structures in the Aspergillus species, such as the arrangement of conidiophores and spore details, appeared visually clearer in images stained with *Beta vulgaris*. However, global image metrics like sharpness and contrast varied across species and, in some cases, were higher for LPCB, indicating that the improved appearance was structure-specific rather than consistent across all samples.

### Statistical tests

The microscopic images were analyzed for different image metrics like sharpness, entropy, contrast, SNR, and edge intensity. Six different microorganisms were tested across different fungi. Bacterial samples stained with *Beta vulgaris* lacked sufficient cellular detail for reliable quantitative analysis. Metrics like sharpness and contrast could not be meaningfully extracted due to the absence of well-defined structures and the predominance of background noise. Therefore, these images were excluded from statistical analysis, and the poor staining performance is described qualitatively. Table 1 shows the different values of these metrics compared with the standard dye.

**Table 1:** Comparison of different image metrics obtained for the various fungi tested for *Beta vulgaris* and standard dyes.

Organism	Dye type	Sharpness	Entropy	Contrast	SNR	Edge intensity
*A. flavus*	*Beta vulgaris*	109.19	6.47	53.9	2.46	0.0053
	LPCB	112.4	6.94	82.11	1.95	0.0034
*A. fumigatus*	*Beta vulgaris*	66.23	6.38	78.58	2.05	0.0077
	LPCB	57.06	6.1	80.83	2.16	0.0039
*A. niger*	*Beta vulgaris*	71.12	7.22	54.66	2.56	0.0101
	LPCB	111.41	7	61.58	2.79	0.0114
*Cryptococcus spp.*	*Beta vulgaris*	62.95	6.34	62.15	2.39	0.0039
	India ink	119	7.34	46.04	2.91	0.0295
*Rhizopus spp.*	*Beta vulgaris*	57.65	6.88	73.43	1.88	0.0103
	LPCB	59.21	7.35	75.36	2.07	0.0058
*Trychophyton spp.*	*Beta vulgaris*	122.76	7.28	69	2.27	0.0537
	LPCB	78.35	7.11	70.45	2.11	0.0053

For the *Aspergillus spp.*, LPCB seems to have an upper hand compared to the *Beta vulgaris* dye. Among them, *Aspergillus niger* had all the image metrics in favor of LPCB, except Entropy. All the images for the *Aspergillus spp.* showed relatively superior contrast values for LPCB compared to *Beta vulgaris*. A similar trend was seen for the *Cryptococcus* and the *Rhizoporus spp*. Sharpness, Entropy, and the SNR were better for LPCB dye compared to *Beta vulgaris*. However, *Beta vulgaris* had better values for Contrast and Edge intensity for the *Cryptococcus spp.* and the *Rhizoporus spp.* respectively. *Beta vulgaris* was more favorable for the *Trychophyton spp.*, with LPCB showing lower values of all the image metrics except contrast.

## Discussion

Natural dyes such as *Beta vulgaris*present a sustainable alternative for microbial staining due to their biodegradable nature and derivation from renewable plant sources. Unlike conventional synthetic dyes that require toxic chemicals and generate hazardous waste, natural dyes minimize environmental pollution and reduce occupational exposure risks in laboratory settings^20^. Their cost-effectiveness and widespread availability also make them particularly suitable for use in resource-constrained environments, offering an accessible and eco-friendly option without significantly compromising staining efficacy. These benefits align with global efforts to promote greener, safer, and more sustainable healthcare practices.

### Gram stain

There was a complete absence of stain uptake in Gram-positive bacteria compared to poor uptake of stain in Gram-negative bacteria. The poor performance of beetroot extract could be due to the thick, complex structure of the cell wall, which is made up of multiple layers of peptidoglycans. These molecules carry negative charges, allowing the basic dyes like safranin to stain. However, the beetroot extract, being acidic (pH 5), could have contributed to its failure to perform as a bacterial stain. This study suggests that aqueous beetroot extract cannot be used for performing simple or Gram staining procedures for bacteria, which is in contrast with findings from^16^. However, with the addition of auxochromes and alteration in pH, the extract can be retried for bacterial staining, which is beyond the scope of the present study.

### Fungal stain

The cell wall and the capsules of most fungi are composed of polysaccharides. These molecules carry a positive charge, which allows acidic dyes like LPCB to stain. Cotton blue is a synthetic dye that is known to cause dermatitis in humans. The mutagenic and carcinogenic potential of the dye is also a known fact. The extract obtained from beetroot is also acidic, having the potential to stain fungi and capsules. The observed difference in staining efficacy between fungi and bacteria can be attributed to the distinct composition of their cell walls. Fungal cell walls are rich in polysaccharides such as chitin and glucans, which present positively charged or polar functional groups that interact favorably with the acidic pigments like betalains in the beetroot extract. These interactions facilitate better dye uptake and retention within fungal structures. The extract, along with pigments, contains some phenolic compounds that help to stabilize the fungal staining and provide additional benefit.

The selective staining ability of *Beta vulgaris* can also be closely related to the chemical nature of its pigments and the structure of microbial cell walls. Betalains in beetroot extract, primarily betacyanins and betaxanthins, are acidic pigments that interact favorably with fungal cell wall components like chitin and glucans through electrostatic and hydrogen bonding mechanisms. These interactions facilitate effective dye binding and visualization of fungal structures. Conventional dyes such as safranin and crystal violet, on the other hand, are basic dyes that bind strongly to negatively charged elements in bacterial cell walls, including teichoic acids and peptidoglycans. LPCB contains synthetic dyes with hydrophobic and ionic interactions designed for fungal structures. These fundamental differences in molecular structure and charge distribution explain why beetroot extract exhibits selective staining affinity toward fungi.

However, the inability of beetroot extract to stain *Candida spp.*, despite being a fungus, may be attributed to its unique cell wall composition and structure. Candida’s cell wall is composed of mannoproteins, glucans, and chitin, but the arrangement may limit the availability of binding sites for acidic pigments such as betalains. Additionally, the capsule-like outer layer of Candida could hinder the penetration of the dye. The mildly acidic pH of the extract (5.5) may further reduce favorable interactions with Candida’s surface components, unlike molds, where the cell walls are more exposed and porous. On the other hand, bacterial cell walls, particularly in Gram-positive species, are composed of thick peptidoglycan layers rich in negatively charged elements that preferentially bind to basic dyes, such as safranin, rather than acidic ones. The extract’s mildly acidic pH (5.5) further promotes binding to fungal cells while limiting interactions with bacterial surfaces, explaining the observed selective staining patterns.

The extract can also be stored for a year at a lower temperature. The extract effectively stains fungi without compromising diagnostic accuracy. Moreover, the bio-degradable, non-hazardous, budget-friendly nature of the beetroot proves it as a better replacement for chemically synthesized dyes.

### Image quality analysis using statistical metrics

To have a meaningful comparison between the *Beta* vulgaris and the standard dye, the mean values of all the statistical metrics were calculated and tabulated in Table 2 along with the mean difference and the standard deviation of the differences. A comparison of the mean values across the different metrics reveals only marginal differences, with the standard dye exhibiting slightly higher values for sharpness, entropy, and contrast. Another important statistical test was to determine the standard deviation of the difference of all the values and to evaluate the effect size via Cohen’s d, which is calculated using Eq. 6.

6 $$\:Cohe{n}^{{\prime\:}}s\:d=\frac{Mean\:Difference}{Standard\:Deviation\:of\:the\:Difference}$$

The Cohen’s d statistic, as shown in Table 2, shows the values in the range of 0.17 to 0.37. A value greater than 0.5 is generally considered to have a moderately significant effect in terms of difference. Since most of the values of Cohen’s d hover around 0.2, showing a very insignificant effect. The interpretation is that the performance of *Beta vulgaris* is not significantly deviating from the results obtained in the standard dye. The value of Cohen’s d, shown as a negative value for edge intensity, only shows that *Beta vulgaris* has performed better than the standard dyes. To further concretize this finding, the Wilcoxon Signed-Rank Test was performed to statistically determine if the *Beta vulgaris* dye deviated significantly in performance compared to the standard test dye. Table 2 also shows the p-value from the Wilcoxon Signed-Rank Test.

**Table 2:** Mean and mean differences of the different image metrics.

Image metric	Sharpness	Entropy	Contrast	SNR	Edge intensity
Mean (*Beta vulgaris*)	81.7	6.8	65.3	2.27	0.015
Mean (Standard dye)	89.6	7.0	69.4	2.33	0.010
Mean difference	7.9	0.2	4.1	0.06	−0.005
Standard deviation of difference	37.0	0.6	14.9	0.36	0.025
$\:Cohen^\prime{s\:d}$	0.21	0.37	0.28	0.17	−0.21
p-value (Wilcoxon Signed-Rank Test)	0.69	0.44	0.31	0.56	0.44

The Wilcoxon Signed-Rank Test yielded p-values of 0.69 for Sharpness, 0.44 for Entropy, 0.31 for Contrast, 0.56 for SNR, and 0.44 for Edge Intensity. None of these p-values were below the conventional threshold for statistical significance (*p* < 0.05), indicating that the observed differences between the two staining approaches are not statistically significant. These results suggest that, within the limitations of this dataset, *Beta vulgaris* dye produces images with comparable quality to those obtained using the standard dye and that it can be considered as a sustainable alternative to the standard.

Despite the absence of statistical significance, it is useful to consider the directional trends in the data. The mean sharpness for *Beta vulgaris* (81.7) was lower than that of the standard dye (89.6), potentially indicating slightly diminished edge definition or focus clarity with the test dye. Similarly, the mean entropy, which is a measure of image complexity and textural variation, was marginally lower for *Beta vulgaris* (6.8) compared to the standard (7.0), which may reflect a slight reduction in visual richness or feature dispersion.

Contrast and SNR values followed a similar pattern, with *Beta vulgaris* yielding slightly lower means (65.3 and 2.27, respectively) compared to the standard dye (69.4 and 2.33). These marginal differences, although consistent in direction, are small in magnitude and subject to variability, as reflected in the non-significant p-values. Interestingly, Edge Intensity, a proxy for localized gradient strength, was marginally higher for *Beta vulgaris* (0.015) than the standard dye (0.010), suggesting a potentially favorable delineation of boundaries with the natural dye, though again, this difference did not reach statistical significance (*p* = 0.44).

In conclusion, while none of the observed differences achieved statistical significance, the results support the feasibility of using *Beta vulgaris* as a substitute for conventional dyes in terms of image quality. The use of global image metrics such as sharpness and contrast may be affected by background noise, debris, or variations in cell density, which could influence the interpretation of staining performance. While segmentation of regions of interest would offer more accurate and targeted analysis, the exploratory scope of this study and variability in fungal morphology across samples presented challenges in implementing a uniform segmentation protocol. Future studies are encouraged to apply advanced segmentation methods to better isolate microbial structures and improve the robustness of image-based assessments.

To cater to staining wide range of microorganisms, other plant extracts may be explored as a complementary stain. The selective staining ability of *Beta vulgaris* suggests that combining it with other plant-based extracts may enhance its utility across a wider spectrum of microorganisms. Various plants, such as turmeric^21^, pomegranate^22–24^, and hibiscus^25^, etc., possess pigments with differing affinities toward microbial structures. Leveraging these differences, future studies could explore combined or sequential staining methods to improve contrast and coverage for both bacterial and fungal diagnostics. Such approaches could contribute to developing a comprehensive, environmentally friendly staining toolkit suitable for diverse clinical and research applications.

## Conclusion

In this work, the potential of *Beta vulgaris* as a natural alternative to conventional staining agents in microbiological applications was investigated. The morphology of fungi was demonstrated more effectively by the beetroot extract compared to the LPCB mount. However, poor staining was observed, particularly for the bacterial stains, where the microscopic images were less sharp and morphologically less defined, even after allowing longer times of application. The quantitative image analysis revealed no statistically significant differences between the natural and standard dyes in terms of sharpness, entropy, contrast, SNR, and edge intensity. These preliminary findings suggest potential trends toward comparable performance for fungal structures, although further validation with larger datasets is necessary to confirm and generalize these observations.

The dye derived from *Beta vulgaris* demonstrated clearer visualization of fungal structures in certain molds, suggesting its potential usefulness as a sustainable alternative for fungal staining in specific research and diagnostic contexts. However, it failed to stain *Candida spp.*, one of the most common clinical yeasts, highlighting the need for further studies to optimize extraction protocols and expand its applicability. Future efforts should concentrate on optimizing the extraction and fixation protocols tailored to *Beta vulgaris* to enhance bacterial staining. Additionally, further studies could expand to multi-organism detection to increase the clinical relevance of the natural dyes.

## Data Availability

Data can be made available from the corresponding author on reasonable request.
